# Rewired Pathways and Disrupted Pathway Crosstalk in Schizophrenia Transcriptomes by Multiple Differential Coexpression Methods

**DOI:** 10.3390/genes12050665

**Published:** 2021-04-29

**Authors:** Hui Yu, Yan Guo, Jingchun Chen, Xiangning Chen, Peilin Jia, Zhongming Zhao

**Affiliations:** 1 Department of Internal Medicine, University of New Mexico, Albuquerque, NM 87131, USA; huiyu1@salud.unm.edu (H.Y.); yaguo@salud.unm.edu (Y.G.); 2 Nevada Institute of Personalized Medicine, University of Nevada Las Vegas, Las Vegas, NV 89154, USA; jingchun.chen@unlv.edu (J.C.); xiangning.chen@unlv.edu (X.C.); 3 Center for Precision Health, School of Biomedical Informatics, The University of Texas Health Science Center at Houston, Houston, TX 77030, USA; pjia@big.ac.cn; 4 Human Genetics Center, School of Public Health, The University of Texas Health Science Center at Houston, Houston, TX 77030, USA; 5 Department of Biomedical Informatics, Vanderbilt University Medical Center, Nashville, TN 37203, USA

**Keywords:** differential co-expression, pathway crosstalk, schizophrenia, PSMC6

## Abstract

Transcriptomic studies of mental disorders using the human brain tissues have been limited, and gene expression signatures in schizophrenia (SCZ) remain elusive. In this study, we applied three differential co-expression methods to analyze five transcriptomic datasets (three RNA-Seq and two microarray datasets) derived from SCZ and matched normal postmortem brain samples. We aimed to uncover biological pathways where internal correlation structure was rewired or inter-coordination was disrupted in SCZ. In total, we identified 60 rewired pathways, many of which were related to neurotransmitter, synapse, immune, and cell adhesion. We found the hub genes, which were on the center of rewired pathways, were highly mutually consistent among the five datasets. The combinatory list of 92 hub genes was generally multi-functional, suggesting their complex and dynamic roles in SCZ pathophysiology. In our constructed pathway crosstalk network, we found “Clostridium neurotoxicity” and “signaling events mediated by focal adhesion kinase” had the highest interactions. We further identified disconnected gene links underlying the disrupted pathway crosstalk. Among them, four gene pairs (*PAK1:SYT1*, *PAK1:RFC5*, *DCTN1:STX1A*, and *GRIA1:MAP2K4*) were normally correlated in universal contexts. In summary, we systematically identified rewired pathways, disrupted pathway crosstalk circuits, and critical genes and gene links in schizophrenia transcriptomes.

## 1. Introduction

Schizophrenia (SCZ) is one of the main mental disorders that disrupt both the physical and the social welfare of the affected subjects and their families. While genome-wide association studies have identified a good number of risk variants to SCZ, functional characterization and neurobiological mechanisms remain to be elucidated [1,2]. For other human diseases, transcriptome profiling of diseased subjects and healthy controls have contributed tremendously to unravel the underlying molecular mechanisms, but such data and corresponding analyses have substantially lagged in schizophrenia studies, mainly because of the difficulty to collect the appropriate human brain tissue. Among the available SCZ transcriptome data, most were tied with microarrays [3,4], a technology gradually replaced by a more powerful approach, RNA-Seq. In recent years, a few studies conducted thorough analyses of SCZ brain transcriptomes, including original data analyses [5,6,7], meta-analyses [8,9], and re-analyses [10,11]. These works suggested probable implication with SCZ of individual biological processes, especially immune system [5,10,12], oxidative stress [11], and cytoskeleton remodeling [7]. Of note, blood samples of SCZ patients have been used to facilitate deciphering molecular neuropathology [13], but most typically, brain transcriptomes were still needed for validating tentative findings [14,15], as SCZ has been commonly considered a brain-related disorder. So far, SCZ and other neuropsychiatric disorders have been considered to have shared polygenic genetic architectures and dysregulated functional modules, while each of them also has unique genetic components [7,16].

Regarding the bioinformatics analysis of such data, we noted that the majority of these original transcriptome studies have benefited from the gene co-expression approach, especially through the application of a popular tool, Weighted correlation network analysis (WGCNA) [17]. By design, WGCNA seeks modules of genes showing correlated expression patterns across experimental conditions and also differential expression between conditions. While it originally targets gene co-expression, WGCNA has been frequently re-purposed to probe into the differential co-expression patterns of individual genes or gene sets, particularly through its later-augmented feature, “module preservation” [18]. As reviewed lately [19], a plethora of new methods directly tackling gene differential co-expression has emerged as additional toolkits for deciphering disease-associated dysregulation mechanisms. Among the numerous differential co-expression tools, a software package “differentially co-expressed genes and links” (DCGL) [20] is capable of identifying differentially co-expressed gene pairs (links) and differentially co-expressed genes, and another software “gene sets net correlations analysis” (GSNCA) [21] commits to evaluating the disruption (rewiring) of internal co-expression within a biologically relevant gene set. Both tools have contributed to a wide range of human disease studies, including many on cancer [22,23,24,25,26] and a few on mental disorders [27,28,29].

Under a specific experimental condition, cellular processes or pathways are coordinated in a particular way to fulfill their programmed functions. Biological pathways are never static and have no definite boundaries in between. The interrogation of context-specific pathway interactions gives rise to pathway crosstalk networks [30,31], which are informative for elucidating pathophysiological mechanisms [32,33,34] and inferring drug efficacy [35,36,37]. We previously inferred a common pathway crosstalk circuit for both SCZ and bipolar disorder through superimposing differentially expressed genes to a protein-protein interaction network [7]. Inspired by the principle of differential network biology [38], we assumed that differentially co-expressed links, such as those detected by computational tool DCGL, may form a network scaffold from which “perturbed” pathway crosstalk circuits can be further identified.

The successful application of differential co-expression approaches in other human disease studies, as well as the intriguing idea of combining differential co-expression networks and pathway crosstalk, has motivated us to carry out the present work of pathway-centric analysis of multiple SCZ transcriptome datasets. Specifically, we investigated disruption of expression correlation within cellular pathways in three RNA-Seq and two microarray datasets, all of which were based on SCZ-vs-control comparison. We performed pathway crosstalk analysis in the scaffold of the gene correlation-loss network, revealing a network of disrupted pathway connections in SCZ. With regard to the SCZ phenotype, our analysis results shed light on internally rewired pathways, important genes as pathway hubs, and disrupted coordination between key signaling pathways.

## 2. Materials and Methods

**Figure 1:**
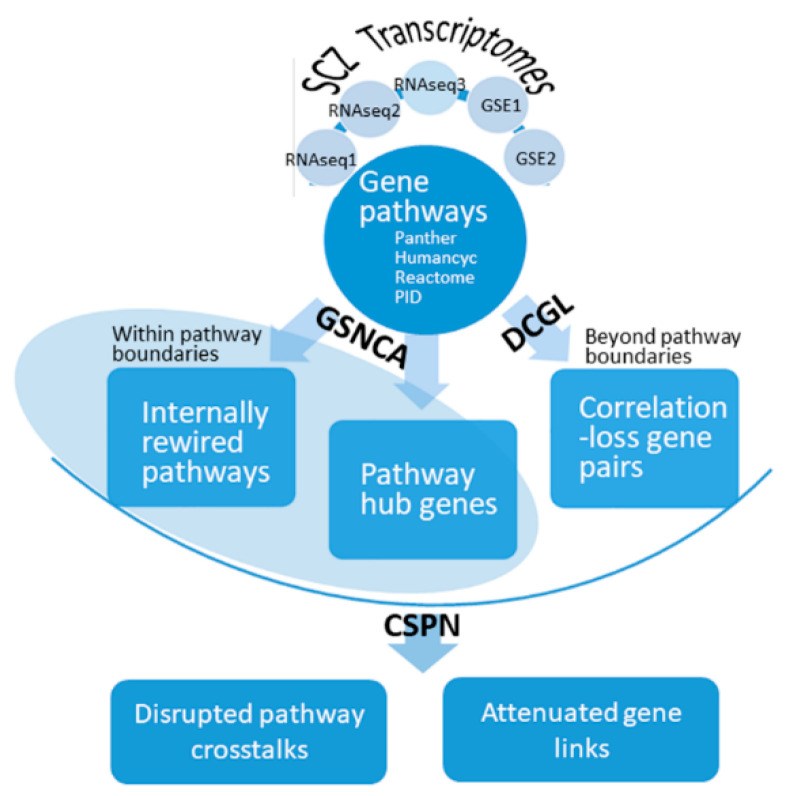
Workflow to identify rewired pathways, disrupted pathway crosstalk circuits, and critical genes and gene links in schizophrenia. A gene expression matrix originating from RNA-Seq or microarray contained expression profiles for tens of thousands of genes across two contrasted phenotypes: control (normal) and schizophrenia (SCZ). Such an expression matrix was analyzed by DCGL to identify correlation-loss gene links, which formed a global gene differential co-expression network. On the other hand, an expression matrix was integrated with gene-to-pathway information to allow GSNCA to distinguish pathways of significant internal co-expression change, as well as hub genes of such pathways. Finally, the three major outputs from GSNCA and DCGL were input to CSPN, which returned disrupted pathway crosstalk circuit and accountable gene links. Five expression data sets were used, and their respective results were mutually compared or complemented in the workflow. SCZ, schizophrenia; DCGL, differentially co-expressed genes and links; CSPN, characteristic sub pathway network; GSNCA, gene sets net correlations analysis.

The workflow started from integrating an SCZ transcriptome dataset with pathway curation knowledge, entailed both intra-pathway and between-pathway differential co-expression analyses, and finally culminated in the identification of schizophrenia-specific pathway crosstalk disruptions and accountable gene correlation losses (Figure 1).

### 2.1. SCZ and Control Transcriptome Datasets

Five RNA-Seq/microarray datasets assaying various human brain regions were included in our study (Table 1). RNAseq1 was generated by us using an RNA-Seq experiment of the anterior cingulated cortex (Brodmann region 24) of postmortem brain samples. This dataset is available upon request to the authors. RNAseq2 and RNAseq3 recorded gene expression profiles in two different brain regions of the same set of donors, and the data were obtained from Stanley Medical Research Institute. These three RNA-Seq datasets corresponded to the discovery and validation datasets underlying our earlier transcriptome study of SCZ and bipolar disorder [7], where more details on alignment and quantification were described. Here, to delimit a set of commonly expressed genes across three RNA-Seq datasets, we required the fraction of non-zero expression values higher than 80% across all samples, and the remaining genes must be common to all three RNA-Seq datasets. As a result, 12,325 genes survived the gene pre-filtering.

GSE1 refers to a microarray dataset GSE21138 downloaded from Gene Expression Omnibus (GEO) for prefrontal cortex brain tissues (Brodmann region 46) [39]. GSE2 refers to another microarray dataset, GSE17612, for prefrontal cortex brain tissues (Brodmann region 10) [40]. Both microarray datasets were generated from the GeneChip^®^ Human Genome U133 Plus 2.0 array. Functions in R packages “hgu133plus2.db” and “rma” were used for data pre-processing. Expression values for probe sets were averaged to the gene level. From the whole set of genes represented on the microarray platform, we retained 11,724 genes that overlapped with the working gene set of the three RNA-Seq datasets.

### 2.2. Data Calibration with Respect to Nuisance Sample Covariates

As illustrated in previous studies [7,39,40], certain sample covariates might have a significant influence on gene expression. To calibrate gene expression values against relevant sample covariates, we fitted the observed expression values of each gene with a linear model, taking into account the variable of primary interest, SCZ vs. control, as well as five nuisance covariates: age, sex, postmortem interval, pH, and cumulative antipsychotic use (due to lack of drug use data, only the first four nuisance covariates were considered in GSE1 and GSE2). In the resultant linear model, when a *p*-value for a coefficient was found less than 0.05, the corresponding covariate was deemed significantly influential on gene expression and the estimated coefficient was subtracted from the gene expression value. The numbers of genes significantly influenced by nuisance covariates were shown in Supplemental File 1: Figure S1. Similar to our previous practice, we found a few hundred to thousand genes being affected per covariate per dataset.

### 2.3. Assessing Differential Co-Expression Levels of Pathways

We downloaded pathways curated in Pathway Commons [41], which assigned 8343 genes to 2191 pathways from four sources (Panther, Humancyc, Reactome, and PID). Of these pathways, only ten were repetitive in more than one source, and we adopted the union of genes across duplicate sources to abolish pathway redundancy. Next, we kept only those genes that appeared in the expression data matrices and dropped the pathways containing four or fewer genes. As a result, we ended up with 1564 and 1561 pathways eligible for analyzing the RNA-Seq and microarray datasets, respectively.

The gene sets net correlations analysis (GSNCA) [21] implemented in R package GSAR was used to assess differential co-expression levels of each candidate pathway. Phenotype labels of samples were permuted 1000 times to obtain *p*-values. An analogous method, GSCA [42], was also implemented and its results were compared to GSNCA results. Given one *p*-value for each pathway out of each dataset, we performed the Fisher’s meta-test to summarize multiple dataset-specific *p*-values, thus making an overall conclusion on each pathway’s differential co-expression significance from all five datasets.

Within one pathway, GSNCA summarizes the expression correlation profile for a gene with respect to all other peer genes, deriving a “weight” index for each gene. Briefly, GSNCA solves the weight vector for all pathway genes under each experimental condition separately and regards the pathways with the most remarkable weight vector changes as the most phenotype-relevant pathways. In one experimental condition, the gene with the highest weight value is designated as the hub gene, around which an intra-pathway gene co-expression network is constructed. The intra-pathway co-expression network is depicted as a union of the first and the second minimum spanning trees, which is identified by minimizing the total path length (sum of correlation distances). By definition, the hub gene of a pathway may not necessarily have the highest degree (i.e., number of connected edges) because it is identified by virtue of the quantitative regulatory importance (i.e., the resolved weight value) rather than the degree [21].

### 2.4. Constructing Gene Differential Co-Expression Networks

We used our R package DCGL (v2.0, Shanghai Center for Bioinformation Technology, Shanghai, China) [43] to identify gene pairs showing changed correlations, i.e., differentially co-expressed links. The Pearson correlation coefficient was adopted as the metric for gene-gene co-expression. Setting the co-expression link density (proportion of co-expressed gene pairs over all possible gene pairs) to 0.01 and a priori differential co-expression rate to 0.1, we obtained ~70 thousand raw differential co-expression links from each dataset, representing an expected fraction of 0.1% for all possible gene-gene links.

Considering that the between-dataset overlapping fraction of differential co-expression links was expected to be 0.1%, the observed overlapping fractions, 0.14% and 0.72% for RNA-Seq datasets and microarray datasets, respectively, were significantly higher than the expected rate (*p* < 0.001, binomial probability model). Microarray datasets presented a higher link overlapping fraction than the RNA-Seq datasets, possibly because of the closer histological relationship of microarray samples compared to the more distantly separated brain regions of the RNA-Seq datasets (Table 1).

The raw differential co-expression links comprised three types, namely “same-signed”, “differently signed”, and “switched”. We found that the same-signed correlation changes were the most prevalent (average fraction was 87.9%) and that gene pairs with positive correlation furthermore dominated the same-signed links (average fraction is 77.6%). Because it was recently reported [44] that a universal pattern of expression decoherence pervades the transcriptome responses to many genetic and environmental perturbations, we decided to analyze only the positive-signed correlation losses, which numbered ~23,500 on average and 117,386 in combination across five datasets. Finally, we took the union set of correlation losses from all five datasets; the merged gene-gene links of correlation losses made up the scaffold network for inferring pathway crosstalk network in the next step.

### 2.5. Inferring Disrupted Pathway Crosstalk Network

Following our previous practices [7,34], here, we employed CSPN [45] to delineate the pathway crosstalk network. CSPN was originally devised to assess pathway-pathway inter-connection based on the frequency of cross-pathway protein-protein interactions. To identify SCZ-disrupted pathway connections in a more straightforward sense, we substituted a network of correlation-loss gene links for the default protein interaction network. Briefly, CSPN requires three major inputs: a network of entities, a subset of entities, and a subset of connections. These three major inputs were indicated in our workflow (Figure 1). We ran CSPN twice, tried two alternative modes separately, and obtained the intersection of significant pathway pairs (*p* < 0.05). The two modes of CSPN considered cross-pathway links in relation to a concerned gene set differently: in the “both” mode, links connecting genes of interest on both ends were considered, while in the “or” mode, links incident to genes of interest were considered.

### 2.6. Venn Diagram and Cellular Component Analysis

Venn diagrams overarching five object lists were rendered with an online tool from Dr. Prof Van de Peer’s Bioinformatics and Evolutionary Genomics group (http://bioinformatics.psb.ugent.be/webtools/Venn/ (accessed on 11/22/2019)). Web service ToppFun [46] (https://toppgene.cchmc.org/enrichment.jsp (accessed on 04/12/2021)) was invoked for examining enriched cellular components of pivotal genes.

## 3. Results

### 3.1. Neural, Immune, and Cell Adhesion Pathways Manifesting Correlation Rewiring

GSNCA [21] evaluates individual biological pathways based on the severity of rewiring of gene co-expression network. At *p* < 0.05, tens or hundreds of significant pathways were selected by GSNCA from each dataset (Table 2, Supplemental File 1: Figure S2). Based on binomial test of overlapping pathways, RNAseq1 showed significant agreement with RNAseq2, GSE1, and GSE2; GSE1 was significantly consistent with RNAseq3 (Table 2).

To further narrow down the pathways to a most relevant set, we identified 60 internally rewired pathways for SCZ (Supplemental File 2: Table S1) as those endorsed by at least two datasets and ascertained with an aggregate p-value of less than 0.01. Of these 60 pathways, seven were accredited by three datasets (Table 3). Apart from the apparent neural pathways “glutamate neurotransmitter release cycle” and “L1CAM interactions”, immune, cell adhesion, and several signaling pathways are featured, consistent with independent findings in peer researches [47,48,49]. Cell adhesion pathways are believed to play a role in neurite outgrowth, growth cone adhesion, and other neural functionalities [50,51,52]. Immune dysregulation was frequently stressed in prior transcriptome studies of mental disorders [8,12,53].

**Figure 2:**
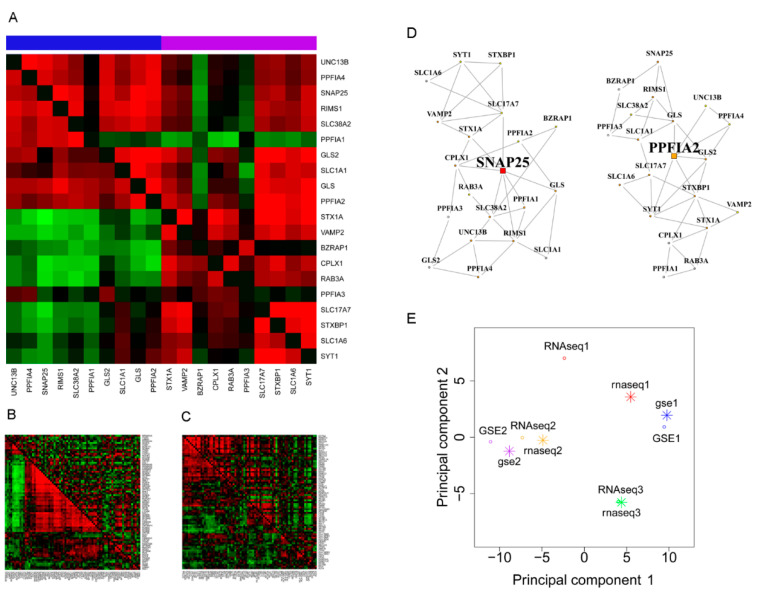
Internal correlation rewiring of pathways perturbed in schizophrenia (SCZ). (**A**–**C**) correlation coefficient heatmaps for three pathways: “glutamate neurotransmitter release cycle” (**A**), “L1CAM interactions” (**B**), and “MHC class II antigen presentation” (**C**). The three heatmaps are derived from expression datasets GSE1, RNAseq3, and RNAseq1, respectively. In each heatmap, the lower triangle and the upper triangle cover gene-gene co-expression level in the control and SCZ groups, respectively. The color bar on top of the heatmap indicates two subgroups of “glutamate neurotransmitter release cycle” genes in the normal condition. (**D**) Gene co-expression networks formed by 20 genes of pathway “glutamate neurotransmitter release cycle” (data source: GSE1). Left, control; right, SCZ. Two highlighted genes (*SNAP25* and *PPFIA2*) are the hub gene in control and SCZ, respectively. Of note, a hub gene does not necessarily have the most connections in the network because it is identified as having the highest regulatory impact (overall co-expression synchrony with all other genes) rather than the highest degree. (**E**) Principal component analysis summarizes pathway rewiring result out of ten separate method-dataset combinations. Uppercase, results from gene sets net correlations analysis (GSNCA); lowercase, results from method gene set co-expression analysis (GSCA). RNAseq1, RNAseq2, RNAseq3, GSE1, and GSE2 are all gene expression dataset names, with details given in Table 1.

In addition to compiling the list of co-expression-perturbed pathways for SCZ, our results can be interpreted for subtle clues to the dysregulation mechanisms within each plausible pathway. From control to SCZ, each pathway may bear a specific disruption pattern of its correlation wiring structure. In “glutamate neurotransmitter release cycle” (Figure 2A), the 20 member genes were naturally divided into two clear-cut groups of high intra-correlations in control samples. Switching from control to SCZ, the intra-group correlations were generally preserved, and some cross-group correlations arose. For “L1CAM interactions” (Figure 2B), a majority of its 75 member genes were engaged in a distinctive correlation clique in control, but this clique faded away in SCZ. For “class II antigen presentation” (Figure 2C), transcriptome correlations were more widespread in SCZ. In addition, visual comparison of intra-pathway co-expression wiring networks for control and SCZ in parallel was enabled, as exemplified for “glutamate neurotransmitter release cycle” (Figure 2D).

As a quality control, we applied an analogous method GSCA [42] to all five datasets and yielded pathway-wise *p*-values in the same manner as GSNCA results. We performed principal component analysis on the 10 lists of *p*-values, resulting from different combinations of method and dataset, to examine the result consistency across datasets and between analysis tools. A higher consistency was seen between the methods than between the datasets (Figure 2E). Specifically, the mean correlation coefficient between the two methods was 0.39, whereas the between-dataset correlation coefficients averaged 0.031 for GSNCA and 0.037 for GSCA.

### 3.2. Dynamic, Pleiotropic Pathway Hub Genes Overrepresented in Neuron Components

Based on the overall regulatory importance of a gene relative to the other genes within the intra-pathway co-expression system, GSNCA designates one gene as the hub of a pathway [21]. For instance, *SNAP25* and *PPFIA2* were hubs of the “glutamate neurotransmitter release cycle” in control and SCZ, respectively (Figure 2D, Table 3). In control, *SNAP25* was found with the greatest co-expression synchrony with all other member genes, whereas in SCZ, such a co-expression center was assumed by *PPFIA2*. The five datasets presented 120, 66, 214, 386, and 45 hub genes for their respective significant pathway lists (Table 4, diagonal cells). Remarkably, every pair of datasets shared a significant fraction of hub genes (all *p* ≤ 0.01 per binomial distribution; Table 4; Supplemental File 1: Figure S2). Similar to our intersection rule for pathways, we took a union of pairwise joint hub genes, leading to a consensus set of 92 genes (Supplemental File 2: Table S2). Because these 92 genes were positioned at the center of SCZ-disrupted pathways, they have termed the pivotal genes hereafter.

**Figure 3:**
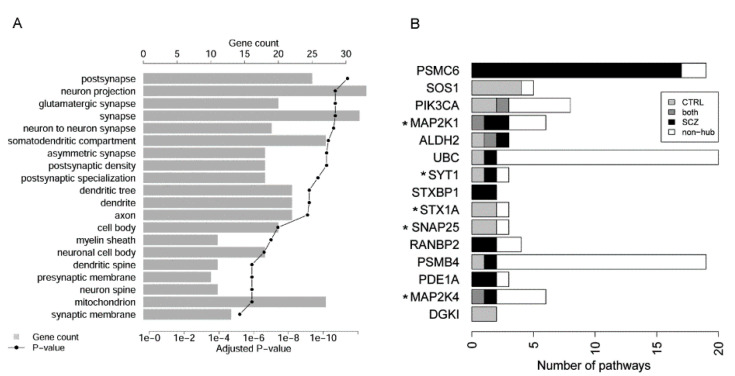
Cellular localization and pathway affiliation of pivotal pathway hub genes. (**A**) Top 20 significantly overrepresented cellular components of 92 pivotal genes. From top to bottom, components were ordered by decreasing statistical significance. (**B**) Number of pathways where a pivotal gene appeared as a hub. Genes were ordered by the number of pathways (colored bar length) where the gene appeared as a hub in either control (CTRL) or SCZ samples. Five gene products localized in “neuron projection” were labeled by asterisk (*).

Applying functional enrichment analysis against Cellular Components of Gene Ontology, we found a plethora of neuron compartments (Figure 3A). For instance, 33 protein products of pivotal genes reside in neuron projection, a significant over-representation ascertained by a Benjamini-Hochberg-adjusted *p*-value of 1.9 × 10^−11^. Other significant neuron cellular localizations included axon, synapse, dendrite, myelin sheath, etc. (Figure 3A) [7].

Despite that we did not take multi-functionality as a defining criterion, we found 45 pivotal genes (48.9%), each participating in two or more SCZ-disrupted pathways. We further identified the respective pathways that each multi-functioning gene showed up as a hub in the control or SCZ samples (Supplemental File 2: Table S2). The results depicted the dynamic hub roles of the 15 most pleiotropic genes (Figure 3B). Of these 15 genes, protein products of *MAP2K1*, *MAP2K4*, *SNAP25*, *STX1A*, and *SYT1* are released to the “neuron projection” compartment. Each of these five genes participates in at least three pathways, and they each appeared as a hub in at least one pathway in healthy brains (Figure 3B, light gray and dark gray bars). From control to SCZ, *SNAP25* and *STX1A* lost their hub roles, *MAP2K1* and *MAP2K4* gained additional hub roles, while *SYT1* manifested the hub role in a different pathway (Figure 3B). Other than these five neuron-located genes, *PSMC6* is of special interest as it gained 17 hub roles in SCZ (Figure 3B, Supplemental File 2: Table S2), becoming the hub of almost all pathways it belongs to (17 out of 19). This proteasome gene was found down-regulated in SCZ [54,55], whereas according to our analysis it seemed to confer an increased activity in SCZ.

### 3.3. Disrupted Pathway Crosstalks Attributed to Gene Correlation Losses

Using DCGL [43], we identified correlation-loss links from five datasets, respectively. The overlapping fraction of raw DCLs across datasets significantly exceeded the random expectation (*p*-value < 0.001, binomial probability model), though the actual number of joint links was technically too small to allow for sizable network inference (Supplemental File 1: Figure S2). Hence, we integrated results from five datasets and constructed a network consisting of all correlation-loss links from individual data sources.

**Figure 4:**
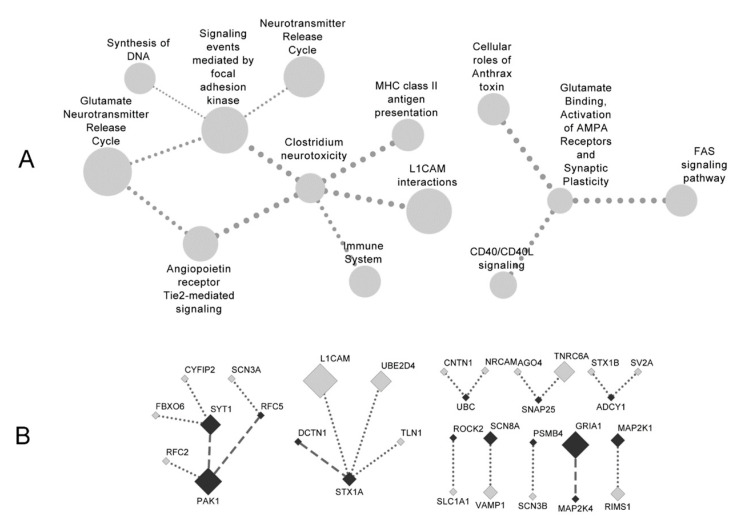
Schizophrenia-specific disrupted pathway crosstalk network (**A**) and accountable correlation-loss gene links (**B**). In (**A**), node size is proportional to the differential co-expression *p*-value (aggregated from five datasets), and edge width is proportional to (disrupted) pathway connection *p*-value. In (**B**), node size is proportional to the multiplicity of genes’ pathway membership; black and gray nodes denote differentiate pivotal genes and other genes, respectively; dashed lines connect pivotal genes on both ends, and dotted lines connect pivotal genes on single ends.

Through appreciating significantly frequent cross-pathway links connecting or incident to pivotal genes (two analysis modes: “both” and “or”), CSPN repeatedly revealed 12 disrupted pathway connections revolving around “Clostridium neurotoxicity” and “signaling events mediated by focal adhesion kinase” (Figure 4A). For verification purposes, we also ran CSPN multiple times on dataset-specific input sets (gene links, hub genes, and correlation-rewired pathways). Of the total 10 trials of different dataset/mode combinations, only three returned sizable result sets (i.e., at least 10 pathway connections), two of which recovered eight and 10 of the 12 formal crosstalk connections, respectively.

The pathway crosstalk disruptions implicated many neuropsychiatric pathways, including neurotransmitter release cycle, clostridium neurotoxicity, L1CAM interactions, among others. Additionally, the network involved focal adhesion and immune pathways, themes of high relevance with SCZ per multiple lines of evidence [8,12,50,51,56,57].

In companion with the disrupted crosstalk of pathway entities (Figure 4A), we presented the disconnected gene links (Figure 4B) underpinning the revealed pathway crosstalk disruptions. We showed 21 correlation-loss gene links covering 14 pivotal hub genes, where four links connected pivotal genes on both ends. According to the reference correlation database CoXpressDB [58], gene pairs *PAK1:SYT1*, *PAK1:RFC5*, *DCTN1:STX1A*, and *GRIA1:MAP2K4* are universally highly correlated (correlation coefficients close to or above 0.30). However, these pivotal inter-connections broke apart in SCZ transcriptomes, accounting for the observed pathway crosstalk disruptions to a large extent.

## 4. Discussion

Due to the ethical standard for the collection of human brain tissue in research, transcriptome data have been very limited for schizophrenia (SCZ), and gene expression dysregulation mechanisms underlying SCZ remain elusive. Here we applied three established differential co-expression methods to analyze three RNA-Seq and two microarray datasets derived from schizophrenia and matched normal samples, representing one of the major efforts to integrate multiple transcriptome data using different computational approaches. In total, we sorted out 60 pathways of internal correlation rewiring (Supplemental File 2: Table S1) by summarizing across five datasets, which encompassed glutamate neurotransmitter release cycle, L1CAM interactions, immune, cell adhesion, and several signaling pathways (FAS, Notch, and GPCR). Joint nomination by multiple datasets resulted in a list of 92 pivotal genes (Supplemental File 2: Table S2) underlying pathway internal rewiring. Finally, we uncovered an SCZ-specific disrupted pathway crosstalk network centered around “Clostridium neurotoxicity” and “signaling events mediated by focal adhesion kinase”, largely attributed to disconnection of four universally correlated gene pairs (*PAK1:SYT1*, *PAK1:RFC5*, *DCTN1:STX1A*, and *GRIA1:MAP2K4*). Put together, we reported SCZ-specific, internally rewired pathways, dynamic pathway hub genes, and disrupted pathway crosstalk connections. These results provide insights into a deep understanding of SCZ pathophysiological mechanisms.

Compared to the conventional differential expression approach, differential co-expression analysis represents a different yet complementary perspective on diseased transcriptomes. Methods purported to identify differentially co-expressed genes, gene connections, and gene sets have been released and improved during the past 15 years [19,59,60]. Nevertheless, differential co-expression analysis methods have not been applied as widely as differential expression analysis methods, and hence they are undergoing rapid developmental advancement. With a rigorous and cautious standard, we imposed analogous methods, tried various parameters, and tested alternative input sets in our workflow. To reassure the findings, robust results were obtained in these repetitive trials, evidence including the significantly high portion of overlapping hub genes and the recurrence of most pathway crosstalk connections from individual datasets. Of note, result consistency across the five datasets was not always as high as expected, especially with regards to the lists of internally rewired pathways. We have applied both GSNCA and GSCA to assess pathway co-expression rewiring, and principal component analysis of the various sets of results supported more evident concordance between the two methods than among the datasets. On the one hand, this result indicated that methodological variants were not a major concern in mining differential co-expression patterns; on the other hand, we were reminded of the heterogeneity arising from different sample sources. In a technical survey of co-expression changes across human tissues, 3–32% of co-expression links were found tissue-specific [61]. It would be ideal if the multiple datasets included in the workflow had originated from the same brain region, but unfortunately, this is very hard to achieve, especially given inadequate data accumulation concerning brain tissues. In our prior transcriptomic study for SCZ and bipolar disorder [7], we recruited data of hippocampus and prefrontal cortex tissues to validate our discoveries out of anterior cingulated cortex samples. In the recent integrative study of five psychiatric disorders [16], microarray datasets originating from diverse brain regions were combined to represent each psychiatric disorder. The authors examined if some results would vary across tissue origins and concluded that the signatures or patterns they discovered were consistent across the four major cortical lobules. In the future, as more postmortem brain transcriptome data are being generated and single-cell RNA-Seq is emerging as a more powerful technology, we expect such data will be used to further interrogate tissue discrepancy across multiple datasets.

Compared with the pathway rewiring results, pathway hub genes manifested a much higher level of consistency across datasets, where every pair of datasets shared a significantly high portion of pathway hubs (Table 4). This could partly be due to the functional multiplicity of many SCZ hub genes; presumably, a small set of pleiotropic genes dictated a great number of pathways, and they might be switching their proprietary pathways across different brain regions or temporal phases. This presumption could explain the low consistency within pathways yet high consistency in hub genes across pathways, and it happens to be an implicit premise underlying most pathway crosstalk analysis strategies [30].

Aggregating numerous gene expression signals to biological processes or functional modules is a common and powerful approach to disease transcriptomes. Analyzing co-expression or differential co-expression of functional gene modules has been a popular approach to dissecting psychiatric disorder’s transcriptomes, which features frequent applications of the well-known software WGCNA or rWGCNA [62]. However, WGCNA is heavily rooted in searching for co-expression modules [19] and neglects certain subtleties of gene correlation [63], and thus, it does not fit well in the framework of differential network biology [38]. In many cases, however, the identified functional modules comprise a large number of constituent genes, presenting a need to further refine the functionally relevant genes to a manageable set. This gap can be filled by the increasing application of pathway crosstalk analysis. Through delineating the significant inter-connections among pathways, this approach highlights the central pathways and their immediate neighbors and characterizes the context-specific inter-coordinates among theme-relevant pathways. Such pathway crosstalk analyses have helped to propose novel pivotal genes for a variety of human diseases [32,33,45]. For example, in an early methodological innovation [64], researchers integrated the differential expression attribute of genes with information on pathway member sharing and showed that groups at the pathway interfaces were more relevant to leukemia subtype distinction. In another example of bioinformatics analysis of hypertensive nephropathy [65], the authors used a functionality through GOSemSim [66] to sketch a function term connection network, whereby they focused attention to genes associated with the bridging terms (terms forming the boundaries of network modules). In the present study, we laid out a differential-network-assisted pathway crosstalk analysis workflow (Figure 1), demonstrating its validity in mining SCZ-relevant rewired pathways, disrupted pathway crosstalk circuits, and critical genes and gene links. This approach with innovative integration of multiple differential co-expression methods proved successful not only in the present SCZ study, but also in a parallel research on chronic kidney disease [67]. Applied on five representative SCZ transcriptome datasets across microarray and RNA-Seq platforms, our analytical framework culminated in a network of pathway crosstalk disrupted in SCZ (Figure 4A), where the pathway dis-coordination is attributed to correlation losses of specific gene-gene pairs (Figure 4B). While the results generated in the present study are modest and are far from a mechanistic demonstration, we hope the pivotal hub genes (Table S2) and the disrupted gene links and pathway connections (Figure 4) would help with generation of biological hypotheses for further validation in future. We are actively refining the proposed analytical framework and also developing a convenient tool to extend the methodological impact to broad fields. In the SCZ domain, new datasets from various sample sources and better qualify data are expected, which will help us improve our analytical framework.

## Figures and Tables

**Table 1:** Basic information of five gene expression matrices.

Dataset	Brain Region	# SCZ Samples	# Control Samples	# Working Genes
RNAseq1 [7]	Anterior cingulated cortex (Brodmann region 24)	31	26	12,325
RNAseq2 [7]	Hippocampus	14	15	
RNAseq3 [7]	Prefrontal cortex	14	15	
GSE1 [39]	Prefrontal cortex brain tissues (Brodmann region 46)	30	29	11,724
GSE2 [40]	Prefrontal cortex brain tissues (Brodmann region 10)	28	23	

**Table 2:** Pairwise overlapping situation of GSNCA-identified pathways among five datasets. Cells in the upper triangle refer to the number of overlapping pathways. Cells in the lower triangle refer to the *p*-values for the corresponding overlapping quantity that was estimated from the binomial distribution model.

	RNAseq1	RNAseq2	RNAseq3	GSE1	GSE2
RNAseq1	105 *	6	12	48	7
RNAseq2	0.029 ^**§**^	44 *	4	9	0
RNAseq3	0.76	0.78	224 *	114	1
GSE1	0.030 ^**§**^	0.99	1.2 × 10^−5,**§**^	58 *	6
GSE2	1.5 × 10^−3,**§**^	1.00	0.95	0.95	32 *

* Number of significant pathways identified from each individual dataset (*p* < 0.05). **^§^** These nominal *p*-values were less than 0.05, and the corresponding false discovery rates were below 0.067.

**Table 3:** Biological pathways showing significant internal correlation structure rewiring in three of the five examined datasets.

Pathway Name	# Genes	Fisher’s Combined *p*-Value	Hub Gene(s) in Controls *	Hub Gene(s) in SCZ *
MHC class II antigen presentation	75	7.0 × 10^−5^	*AP1S1, CLTC, KIF3B*	*DCTN1, KIFAP3, SPTBN2*
Synthesis of epoxy (EET) and dihydroxyeicosatrienoic acids (DHET)	5	4.0 × 10^−4^	*CYP1A2, EPHX2*	*CYP1B1, CYP2J2, EPHX2*
Glutamate neurotransmitter release cycle	20	0.0015	*SNAP25, RAB3A, SYT1*	*PPFIA2, STXBP1*
L1CAM interactions	75	0.0019	*CHL1, DLG1, SCN8A*	*CHL1, DLG3, MAP2K1*
GPCR downstream signaling	258	0.0037	*DGKI, SOS1, PRKCE*	*PDE1A, PRKCE, RGS7*
Receptor-ligand binding initiates the second proteolytic cleavage of Notch receptor	13	0.0037	*ADAM10, NOTCH3, NOTCH4*	*NOTCH1, NOTCH3, UBC*
a6b1 and a6b4 Integrin signaling	32	0.0050	*PIK3CA, YWHAG*	*PIK3CA, YWHAB, YWHAG*
G alpha (i) signaling events	97	0.0056	*ADCY1, CHRM4, GNG13*	*GNG11, GNG13, RGS7*
FAS signaling pathway	18	0.0056	*CYC1, GSN, MAP2K4*	*LMNB2, MAP2K4, MAPK9*

* The hub genes nominated by different datasets may not be the same, and they were all shown.

**Table 4:** Pairwise overlapping of GSNCA-identified pathway hub genes among five datasets. Diagonal: number of hub genes merged from dataset-specific significant pathways. Cells in the upper triangle denote the numbers of overlapping hub genes. Cells in the lower triangle denote the *p*-values for the corresponding overlapping quantity that was estimated from the binomial distribution model.

	RNAseq1	RNAseq2	RNAseq3	GSE1	GSE2
RNAseq1	120 *	7	18	25	3
RNAseq2	4.84 × 10^−7^	66 *	7	11	3
RNAseq3	9.24 × 10^−13^	2.76 × 10^−5^	214 *	31	4
GSE1	2.12 × 10^−14^	1.52 × 10^−6^	1.25 × 10^−12^	386 *	11
GSE2	1.25 × 10^−3^	1.29 × 10^−4^	1.53 × 10^−3^	5.24 × 10^−8^	45 *

* Number of hub genes of correlation-rewired pathways identified from each individual dataset.

## Data Availability

Not applicable.

## Supplementary Materials

The following are available online at https://www.mdpi.com/article/10.3390/genes12050665/s1, Figure S1: Numbers of genes whose expression values were significantly influenced by nuisance covariates, Figure S2: Venn diagrams show overlapping of significant pathways (A), hub genes (B), and correlation-loss links (C) among five expression datasets, Table S1: Sixty co-expression-perturbed pathways for SCZ, Table S2: Ninety-two pivotal genes.

## Abbreviations

CSPN	Characteristic sub pathway network
DCGL	Differentially co-expressed genes and links
GSCA	Gene set co-expression analysis
GSE1	A microarray gene expression dataset of prefrontal cortex brain tissues (Brodmann region 46)
GSE2	A microarray gene expression dataset of prefrontal cortex brain tissues (Brodmann region 10)
GSNCA	Gene sets net correlations analysis
RNAseq1	An RNA-Seq dataset of anterior cingulated cortex tissues (Brodmann region 24)
RNAseq2	An RNA-Seq dataset of hippocampus tissues
RNAseq3	An RNA-Seq dataset of prefrontal cortex tissues
SCZ	Schizophrenia
