# Supplementary material for: Rewired Pathways and Disrupted Pathway Crosstalk in Schizophrenia Transcriptomes by Multiple Differential Coexpression Methods
Source: Genes (Basel). 2021 Apr 29;12(5):665. doi: 10.3390/genes12050665 (PMC8146818; doi:10.3390/genes12050665)
Supplement: Supplementary file 1 [file genes-12-00665-s001.zip › supplementary/SuppFile - Figures.docx]

Supplementary Figures

**Rewired Pathways and Disrupted Pathway-crosstalks in Schizophrenia Transcriptomes by Multiple Differential Coexpression Methods**

**Hui Yu et al.**


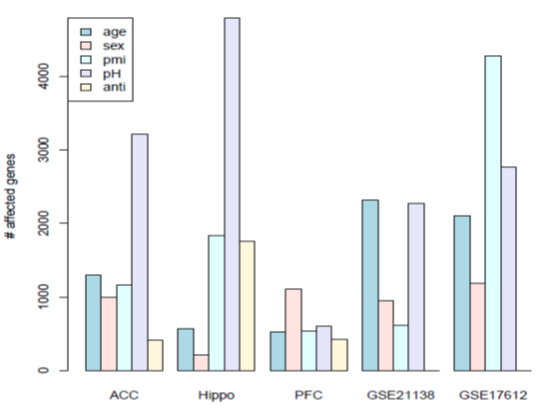


**Figure S1.** Numbers of genes whose expression values were significantly influenced by nuisance covariates. The observed expression values of each gene were fitted with a linear model that accounted for six variables: SCZ vs. control (variable of major interest), age, sex, post-mortem interval, pH, and cumulative antipsychotic use. Due to lack of drug use data, only the first four nuisance covariates were considered in data processing for datasets GSE1 and GSE2. A few hundred to thousand genes being affected per covariate per dataset. Most covariates showed impact on fewer genes in the RNA-Seq datasets than in microarray datasets.


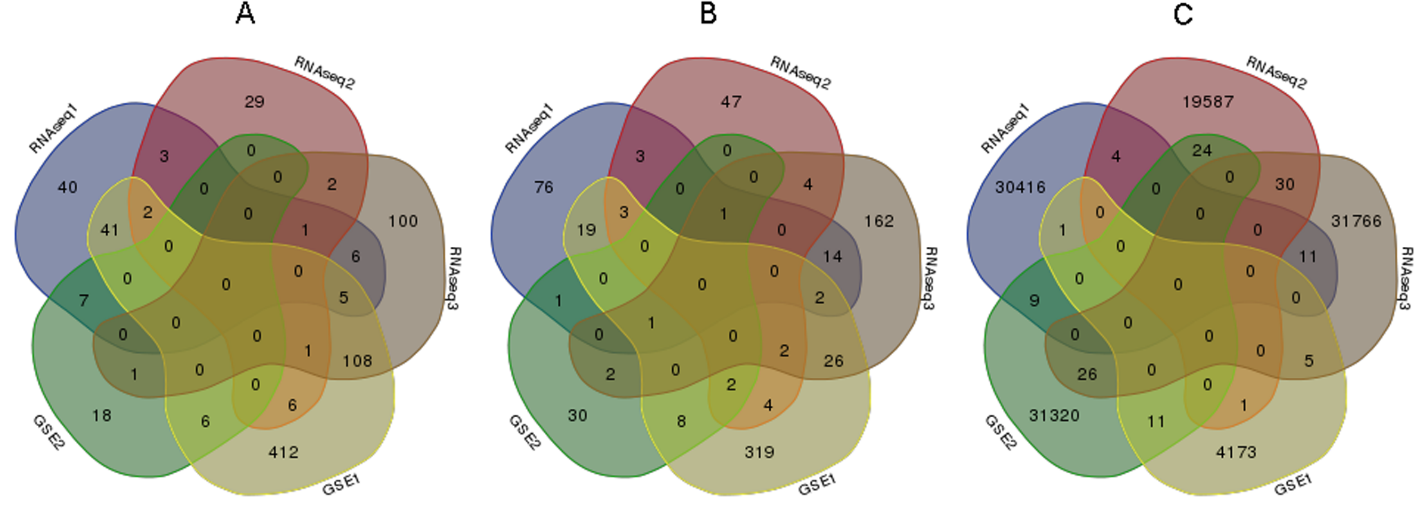


**Figure S2.** Venn diagrams show overlapping of significant pathways (A), hub genes (B), and correlation loss links (C) among five expression datasets. (A) by setting GSNCA [[1](#_ENREF_1)] p value threshold at 0.05, tens or hundreds of significant pathways were identified from dataset (Table 2, diagonal numbers). (B) Intra-pathway hub genes of significant pathways from five datasets. (C) The five datasets returned 30,441 (RNAseq1), 19,647 (RNAseq2), 31,839 (RNAseq3), 4,191 (GSE1), and 31,390 (GSE2) correlation loss links, with an average of ~23.5K links and 117,386 links in combination (unique ones). Four out of ten pairwise overlap portions in A were significantly larger than random expectation (Binomial model p<0.05); all pairwise overlap portions in B and C were significantly larger than random expectation (Binomial model p<0.01). More details pertaining to subfigures A and B are available in Tables 2 and 4.
